# Wound Infection

**Published:** 1916-07

**Authors:** 


### Reviews of Books.

**Wound Infection.** By Col. Sir ALMROTH E. WRIGHT, M.D., F.R.S. Pp. 96. University of London Press Ltd. 1915. Price 2s. 6d.

This little book is not the latest pronouncement of Sir Almroth Wright on this subject—that was an address at the Royal Society of Medicine in the autumn, which has since been published in the medical journals [1]—but it is an address given at the same place earlier in the year. The author has done a great deal of most interesting work in studying the pathology of wound infection and Nature's resistance to it at his laboratory at Boulogne, and the account he gives in this book of his experiments and observations with regard to the emigration of leucocytes is of much interest; but since this little book of Sir A. Wright was published his deductions from these experiments, and his views as to the method by which the application of saline to a wound causes an increased flow of lymph, and the value of the lymph as an agent for the destruction of micro-organisms, have been adversely criticised by Sir Watson Cheyne in his *British Journal of Surgery* for January, 1916, and by Dr. Parry Morgan in a paper read before the Pathological Section of the Royal Society of Medicine (*Proceedings Royal Society of Medicine*, 1916, vol. ix. Path. Section). Dr. Parry Morgan was a worker at Sir A. Wright's laboratory at Boulogne. Sir Almroth Wright's deductions have also been criticised by Dr. Kenneth Taylor in the *British Medical Journal* for Sept. 2nd, 1916, in a paper on "The Mechanism of Saline Dressings."

[1] Since this review was written, another paper has been published in the journals on June 3rd, but it is only a summary of the treatment he recommends for gunshot wounds.

We strongly dissent from his teaching with regard to the uselessness of antiseptics. Let us examine the grounds on which he maintains they are useless. He admits that antiseptics are useful in the compound fractures of civil life because " here the microbes lie exposed on the external surface of the bone which has been thrust through the skin," but not in gunshot wounds, in which he says " they have been carried down deep into the tissues and lie on the inner face of a torn and ragged track, and that track is blocked by blood-clot and hernia of muscles." Now the description he gives of a gunshot wound is really the condition we have to deal with when we disinfect many compound fractures in civil life. It is not simply a protruding bone we have to disinfect, but extensive laceration within the limb. Yet in many cases, by the thorough cleansing of the wound and the application of a strong antiseptic, we are able to sterilise such a wound in civil practice. We are quite ready to admit that some very extensive and tortuous gunshot wounds may be most difficult to sterilise, but we cannot accept Sir Almroth Wright's description of the ease with which the compound fractures of civil life can be sterilised.

The next objection raised by the author to the value of antiseptics is that the pus in the wound will greatly hinder their action. But the chief use of antiseptics is to sterilise the wound before pus is formed. We know that serum—which Sir A. Wright says has less " quenching effect " on the antiseptic than pus—acts chemically on most antiseptics, so as to considerably reduce their power. But it is only a question of the use of an abundant quantity, and of prolonged application. The author then asks the question : " Is there any reasonable prospect of sterilising the wound by an application of antiseptics ? " In his answer he considers only suppurating wounds. But as we have just pointed out, it is to prevent suppuration occurring that an antiseptic is of the greatest value. He says antiseptics cannot reach the organisms in the granulation of the wound. But it should be our object to apply the antiseptic most thoroughly to the wound before the organism has penetrated its wall, and before granulation tissue is found, and the experiments related by Sir Watson Cheyne in his paper in the *British Journal of Surgery* (*ibid.*) seems to prove that antiseptics do penetrate the living tissues. Moreover, Carrel says that by the prolonged use of the hypochite of soda he can sterilise suppurating wounds.

Now, although Sir Almroth Wright tries to show what useless things antiseptics are, yet curiously enough he says that they have value in preventing the spread of infection from one septic wound to another, and therein he considers lies their whole value. But of their value for this purpose, used as a douche to the wound, we are very doubtful. The washing out of a septic wound with an antiseptic, which is the method by which he considers it has prevented infection spreading to other wounds, seems to us a futile proceeding. What is really required to prevent the spread of infection is the boiling of all instruments, the wearing of sterilised rubber gloves, and the sterilising, by boiling if possible, of all bowls.

The author wonders why the "obvious non-success of the antiseptic treatment is not generally appreciated." We hope since this was written he has made some observations as to the great success of the antiseptic treatment in the hands of Dr. Alexis Carrel, in preventing wound infections among the recently-wounded French soldiers, and has noticed how well wounds already suppurating have progressed under antiseptic treatment in the hands of many surgeons. What can he show as the result of his "physiological" methods? Can he induce Nature to sterilise a badly-infected wound, however freely lymph is poured out? If the "non-success of the antiseptic treatment is not generally appreciated," the non-success of the "physiological" ought to be. His experiments with regard to emigration of leucocytes, and the action of pus on antiseptics, are no doubt very interesting, but his determined opposition to the use of antiseptics has retarded, not advanced progress, in dealing with the wounds of war.

Sir Almroth Wright proposes to treat infected wounds with vaccines. This does not seem reasonable. The wound is already swarming with organisms, and if their presence there cannot induce such chemical changes in the serum as will lead to the production of immunity, how can he expect a comparatively small number of dead organisms injected into the tissue to do so? If the chemical products of the organism in the wound cannot reach the blood, as the vaccine can, then how can the products of the action of the vaccine reach the tissues in which the microbes lie, so as to produce a defensive action in those tissues? But a truly prophylactic use of the vaccines, as in typhoid fever—*i.e.*, before the infliction of the wound—might be a great help in preventing infection, or in reducing its virulence, and, as has been suggested by Sir Watson Cheyne, this might be carried out during the training of the recruit.

In his epilogue the author makes a very remarkable suggestion. It is no less than a recommendation that those who treat the wounded should do so not as they think best, but on lines laid down by some authority, acting on the advice of a committee of Physicians, Surgeons and Pathologists. The idea of treating patients as a machine would, is so startling that it almost takes one's breath away. Suppose the treatment forced on the military practitioner by the authority turned out to be a mistake, and it was found to have been unreasonable and unsuccessful in the light of further investigation and experience, would it be considered an infringement of military discipline to criticise it? If the author converted all the members of the committee to his view, and the authority laid it down that all antiseptics were to be put down the drain, and all wounds alone treated by the "physiological" method, would the remarks made in this review be regarded as a heresy demanding stern measures of suppression?

